# Variability of *EGFR* exon 20 insertions in 24 468 Chinese lung cancer patients and their divergent responses to EGFR inhibitors

**DOI:** 10.1002/1878-0261.12710

**Published:** 2020-06-15

**Authors:** YanRu Qin, Hong Jian, Xiaoling Tong, Xue Wu, Fufeng Wang, Yang W. Shao, Xinmin Zhao

**Affiliations:** ^1^ Department of Oncology The First Affiliated Hospital of Zhengzhou University Zhengzhou China; ^2^ Shanghai Lung Cancer Center, Shanghai Chest Hospital Shanghai Jiao Tong University Shanghai China; ^3^ Translational Medicine Research Institute Geneseeq Technology Inc Toronto ON Canada; ^4^ Nanjing Geneseeq Technology Inc. Nanjing China; ^5^ School of Public Health Nanjing Medical University Nanjing China; ^6^ Department of Medical Oncology Fudan University Shanghai Cancer Center Shanghai China; ^7^ Department of Oncology, Shanghai Medical College Fudan University Shanghai China

**Keywords:** EGFR exon 20 insertion, lung cancer, osimertinib, poziotinib, tyrosine kinase inhibitor

## Abstract

*EGFR* exon 20 insertions (*EGFR* e20ins) account for up to 10% of *EGFR* mutations in lung cancer; however, tumors with *EGFR* e20ins had poor response rates to EGFR tyrosine kinase inhibitors (TKIs) including gefitinib, erlotinib, afatinib, and osimertinib, and the heterogeneity of *EGFR* e20ins further complicates the clinical studies. Here, we retrospectively screened next‐generation sequencing (NGS) data from 24 468 lung cancer patients, and a total of 85 unique *EGFR* e20ins variants were identified in 547 cases (2.24%), with p.A767_V769dup (25.1%) and p.S768_D770dup (17.6%) being the most prevalent ones. Comprehensive genomic profiling revealed that *TP53* mutations frequently coexisted with p.H773dup (77.8%, *P* = 0.0558) and p.A767_V769dup (62.8%, *P* = 0.0325), while *RB1* mutations usually co‐occurred with p.H773_V774insAH (33.3%, *P* = 0.0551), implying that different *EGFR* e20ins variants might require distinct genomic context for tumorigenesis and/or maintenance. Despite that treatment regimens were highly diverse for *EGFR* e20ins‐positive patients, we observed an overall response rate of 14% and a disease control rate (DCR) of 38.4% in 65 patients who received at least one EGFR TKI. The progression‐free survival (PFS) differs significantly in six representative *EGFR* e20ins variants (*P* = 0.017), and *EGFR* p.A763_Y764insFQEA was associated with better PFS than other *EGFR* e20ins when treating with various EGFR TKIs. Some *EGFR* e20ins variants showed at least partial response to first‐generation EGFR TKIs, including p.A767_V769dup, p.S768_D770dup, p.N771_H773dup, p.A763_Y764insFQEA, and p.D770_N771insG. Poziotinib achieved higher DCR for p.S768_D770dup than for p.A767_V769dup, whereas osimertinib showed limited effects for these two insertions when used as the first‐line treatment. Overall, our results demonstrated that *EGFR* e20ins were highly diversified in terms of insertion patterns and co‐occurring mutations and these *EGFR* e20ins variants showed different clinical responses to various EGFR TKIs, suggesting the clinical importance of selecting proper EGFR TKI treatment based on the specific *EGFR* e20ins type.

## Graphical abstract

*EGFR* exon 20 insertions (*EGFR* e20ins) were highly diversified in terms of insertion patterns and co‐occurring mutations, and these *EGFR* e20ins variants showed different clinical responses to various EGFR tyrosine kinase inhibitors (TKIs) in lung cancer, suggesting the clinical importance of selecting proper EGFR TKI treatment based on the specific *EGFR* e20ins type.

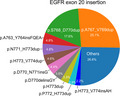

**Abbreviations**
CNV: Copy number variations
DCR: Disease control rate
EGFR e19del: EGFR exon 19 deletion
EGFR e20ins: EGFR exon 20 insertions
FFPE: Formalin‐fixed paraffin‐embedded
MAF: Mutant allele frequency
NSCLC: Non‐small‐cell lung cancer
ORR: Overall response rate
PFS: Progression‐free survival
PR: Partial response
SD: Stable disease
TKI: Tyrosine kinase inhibitor

## 1 Introduction

*EGFR* is one of the most commonly mutated genes in lung cancer patients, especially in Asia where an incidence of 40–60% was observed (Jain *et al*., 2015; Liu *et al*., 2017). Two dominant *EGFR* mutations include L858R and exon 19 deletion (e19del) that result in constitutive kinase activation, and for which multiple ATP‐competitive reversible and irreversible EGFR tyrosine kinase inhibitors (TKIs) are available. Other, less common, EGFR‐activating mutations, including the point mutations, G719X and L861Q, as well as in‐frame exon 19 insertions also produce EGFRs that are sensitive to TKI treatment (He *et al*., 2012; Iyevleva *et al*., 2014; Kobayashi *et al*., 2015). However, in‐frame insertions at exon 20 of *EGFR* (*EGFR* e20ins), which account for ~ 4–10% of *EGFR* mutations, have generally been reported with poor responses to the first‐generation EGFR inhibitors, gefitinib and erlotinib (Beau‐Faller *et al*., 2014; Naidoo *et al*., 2015), and second‐generation inhibitor, afatinib (Yang *et al*., 2015). Sporadic case studies reported the likely response of such *EGFR* e20ins mutants to the third‐generation TKI, osimertinib (Fang *et al*., 2019a; Piotrowska *et al*., 2018b; van Veggel *et al*., 2020), and the Hsp90 inhibitor, luminespib (Jorge *et al*., 2018).

The clinical characteristics of patients with *EGFR* e20ins are similar to those with *EGFR* classic mutations, which are more prevalent in females, nonsmokers, and tumors with adenocarcinoma histology (Oxnard *et al*., 2013; Riess *et al*., 2018). *EGFR* e20ins are generally 3–21 base pairs in length (corresponding to 1–7 amino acids) at different locations of *EGFR* exon 20, and in particular, immediately on the C‐terminal side of the αC helix. The crystal structure is only available for EGFR D770insNPG and confirmed the autoactivation state by an altered αC helix orientation that destabilizes the inactive state of the kinase (Yasuda *et al*., 2013). However, the heterogeneity of the insertion length and position increases the difficulty of generating consistent and comparable conclusions from separate clinical studies, since different insertions result in unique kinase activities and responses to treatments (Ikemura *et al*., 2019; Yasuda *et al*., 2013). Currently, chemotherapy is a standard therapy for lung cancer patients with *EGFR* exon 20 insertions due to the limited clinical benefits of TKI treatments observed in previous studies (Byeon *et al*., 2019). Here, we present the largest population study of *EGFR* e20ins with the clinical use of multiple EGFR TKIs in a subset of patients.

## 2 Materials and methods

### 2.1 Patients and samples

Lung cancer cases carrying *EGFR* e20ins were extracted from an internal database at Geneseeq Technology Inc., China, which contains tumor DNA sequencing data of 24 468 lung cancer patients. The selected 547 cases contained 375 formalin‐fixed paraffin‐embedded (FFPE) sections, 145 plasma, and 27 pleural effusion samples, which were used for targeted sequencing of 139 lung cancer‐related genes. All samples were matched to a whole‐blood sample from the same patient as a control to identify germline mutations. DNA extraction and sequencing library preparation followed the protocols described previously (Fang *et al*., 2019b; Yang *et al*., 2018). All samples were tested in a Clinical Laboratory Improvement Amendments‐ and College of American Pathologists‐certified genomic testing facility (Nanjing Geneseeq Technology Inc., Nanjing, China). Different types of genetic alterations were called using an internally validated bioinformatics analysis pipeline (Tong *et al*., 2019). For calling of copy number variations (CNV), we used an in‐house developed bioinformatics pipeline to analyze CNV and the pipeline has been validated in 38 samples against their droplet digital polymerase chain reaction results as ‘gold standard’. The system noise in copy number data was reduced by principal component analysis of 100 normal samples sequenced in the same batch. A fold change of ≥ 1.6 and ≥ 2.0 is used to detect CNV gain in liquid biopsy samples and tumor tissues, respectively, while a fold change ratio ≤ 0.6 is used to detect CNV loss in both sample types. Clinical information, including age at diagnosis, sex, disease stage, and treatment history, was extracted from the medical records provided by physicians during the service order or clinical follow‐ups during the treatment course. Tumor responses were classified according to the response evaluation criteria for solid tumors (RECIST 1.1). Disease control status is defined as the ‘best response status to date’, which includes complete response, partial response (PR), and stable disease (SD), and disease control rate (DCR) at 8 weeks is used for analysis. Informed written consent was obtained from each patient at the time of sample submission. The study methodologies conformed to the standards set by the Declaration of Helsinki and was approved by the ethics committee of Fudan University.

### 2.2 Data analysis and statistics

All statistical tests were conducted in r version 3.6.1, https://cran.r‐project.org/bin/windows/base/old/3.6.1/. The Kaplan–Meier method was used to calculate survival rates, and the log‐rank test was used to analyze differences between the groups. A statistically significant difference was set as *P* < 0.05. The chi‐square test was used to compare the DCRs among different TKI groups. Fisher’s exact test was used to analyze the concurrency of different gene mutations among different EGFR e20ins subtypes.

## 3 Results

### 3.1 Clinical characteristics and variations types of EGFR e20ins

Massively parallel sequencing of tumors or liquid biopsy samples from 24 468 patients with lung cancer identified a total of 547 cases (2.24%) with *EGFR* e20ins mutations. The median age of patients at the time of diagnosis was 59 years old, with a range of 28–96 years old (Table 1). A total of 52.7% (*n* = 288) of patients were female, which was slightly more than the number of male patients (45.5%, *n* = 249). The sex of ten patients was unknown (1.8%). Most patients had non‐small‐cell lung cancer (NSCLC), including adenocarcinoma (77.9%, *n* = 426) as the major histological type, which was much higher than the frequency of squamous (2.0%, *n* = 11) and adenosquamous carcinomas (2.0%, *n* = 11).

**Table 1:** Clinical characteristics of patients with EGFR exon 20 insertions. Some patients received multiple lines of TKI treatment.

Characteristics	Value or no. of patients (%)
Age of diagnosis (year)	
Median (range)	59 (28–96)
≤ 45	67 (12.2)
45–60	200 (36.6)
61–70	161 (29.4)
≥ 70	69 (12.6)
NA	50 (9.1)
Gender	
Male	249 (45.5)
Female	288 (52.7)
NA	10 (1.8)
Clinical stage	
I–II	15 (2.7)
III	18 (3.3)
IV	155 (28.3)
NA	359 (65.6)
Histology classification	
Adenocarcinoma	426 (77.9)
Squamous	11 (2.0)
Mixed	9 (1.6)
NA	101 (18.5)
Patients experience EGFR TKI treatment (*n* = 65)	
1st‐gen TKIs	51 (9.3)
Afatinib	10 (1.8)
Poziotinib	12 (2.2)
Osimertinib	22 (4.0)

**Fig. 1:**
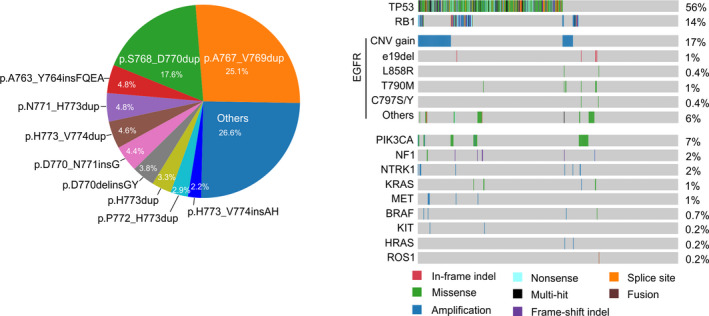
Frequencies of different *EGFR* e20ins and other genetic alterations in patients with *EGFR* e20ins. (A) Pie chart showing the frequency of the most common *EGFR* e20ins. (B) Comutation plot illustrates genetic alterations in *EGFR* and other primary driver genes.

A total of 85 unique *EGFR* e20ins were identified, and each patient had only one insertion. Most insertions were 1–3 amino acids in length (six amino acids at most), and the most frequent insertions were p.A767_V769dup (25.1%, *n* = 145) and p.S768_D770dup (17.6%, *n* = 96, Fig. 1A). A total of 94.7% of insertions occurred in the EGFR loop region starting from the p.A767 residue located immediately on the C‐terminal side of the αC helix and were highly diversified in three different formats: insertions, deletion–insertions (delins), and duplications (Fig. S1).

In addition to *EGFR*, the mutation concurrence in 13 other clinical relevant driver genes was inspected, including *ALK*, *MET*, *KRAS*, *ERBB2*, *ROS1*, *RET*, *BRAF*, *HRAS*, *NF1*, *MEK1*, *AKT1*, *PIK3CA,* and *PTEN* (Pao and Girard, 2011). Only variations in those genes that were interpreted as pathogenic or likely pathogenic by ACMG/AMP 2015 guidelines (Richards *et al*., 2015) were demonstrated (Fig. 1B). Apart from the well‐characterized alterations in *EGFR*, *KRAS,* and *PIK3CA,* we observed gain‐of‐function alterations including copy number gain in *BRAF*, *MET, HRAS,* and *KIT,* and loss of function in *NF1*. Genetic characterizations of *EGFR* e20ins patients revealed that *EGFR* amplifications were present in 17% (*n* = 91), while the concurrence of *EGFR* L858R (0.4%, *n* = 2) and e19del (1%, *n* = 5) was rare (Fig. 1B). Secondary *EGFR* mutations, including T790M and C797S/C, were also identified in nine patients who underwent *EGFR* TKI treatment. Mutations in two common tumor suppressors, *TP53* and *RB1,* were observed in 56% and 14% of patients, respectively. Other driver mutations with evidence of pathogenicity occurred primarily in oncogenes at a very low frequency, and in a mutually exclusive pattern (Fig. 1B). *PIK3CA* (7%) was the most frequently activated oncogene, while *NF1* (2%) was the most frequently deactivated tumor suppressor. With a few exceptions, the mutation frequencies of these critical genes were comparable among different *EGFR* e20ins (Table S1). The concurrence of tumor suppressors *TP53* and *RB1* was also investigated. *TP53* mutations were most prevalent in patients with *EGFR* p.H773dup (77.8%, *P* = 0.0558) and p.A767_V769dup (62.8%, *P* = 0.0325), while *RB1* mutations were most abundant in p.H773_V774insAH (33.3%, *P* = 0.0551) patients. Moreover, we observed the highest co‐occurrence of non‐*EGFR* driver mutations in patients with p.H773_V774insAH (33.3%, *P* = 0.0596) and p.H773_V774dup (28%, *P* = 0.0342). The coexistence of such alterations potentially shaped tumor responses to different EGFR TKI treatments.

### 3.2 Distinct responses of different *EGFR* e20ins to different EGFR TKI treatments

Seventy patients (12.8%) received EGFR TKI treatment, and 19 (3.5%) were treated with more than one type of TKI. The first‐generation TKIs (*n* = 51) were the most commonly used, including gefitinib/erlotinib in 38 patients and icotinib in 15 patients. The third‐generation TKI, osimertinib, was also used in 22 patients, but mostly as the treatment following first‐line TKIs (Fig. S2). Five patients were identified with *EGFR* L858R or e19del simultaneously and responded to TKI treatment. Therefore, those patients were excluded from subsequent analyses. In the remaining 65 patients, the overall response rate (ORR) was low for the first‐generation TKI (1st‐gen TKI, erlotinib/gefitinib/icotinib) treatment (ORR = 12.8%), and comparable to afatinib (11.1%), while osimertinib (16.7%) and poziotinib (16.7%) were higher (Table 2).

**Table 2:** Treatment effects of TKIs in different EGFR e20ins. 1st‐gen TKIs include gefitinib, erlotinib, and icotinib. PD, progressive disease.

EGFR e20ins (No. of TKI‐treated patients)	1st‐gen TKIs	Afatinib	Osimertinib	Poziotinib
A767_V769dup (*n* = 17)	2 PR, 3 SD, 7 PD		1 PR, 2 PD	1 SD, 3 PD
S768_D770dup (*n* = 14)	3 SD, 8 PD	1 SD, 3 PD	1 SD, 1 PD, 1 PD	2 SD, 1 PR, 1 PD
N771_H773dup (*n* = 5)	1 SD, 2 PD	1 SD	1 SD, 1 PD	
A763_Y764insFQEA (*n* = 5)	3 PR, 3 SD		1 PR, 1 PD	
P772_H773dup (*n* = 3)	2 PD		1 PD	
H773_V774dup (*n* = 3)	1 PD	1 SD, 1 PD	1 PD	1 PD
H773dup (*n* = 3)	1 PD	1 SD		1 PD
D770delinsGY (*n* = 2)	3 PD		1 PD	
D770_N771insG (*n* = 2)	1 PR, 1 SD			
D770_N771insGL (*n* = 1)			1 PD	
D770_N771insY (*n* = 1)	1 PD		1 PD	
N771_P772insT (*n* = 1)				1 PR
H773_V774insAH (*n* = 1)			1 PR	
N771_P772insHN (*n* = 1)	1 PD		1 SD	
N771_P772insL (*n* = 1)			1 SD	
N771delinsTH (*n* = 1)	1 PD			
P772_V774dup (*n* = 1)	1 PD			
V769_D770insGTV (*n* = 1)	1 PD			
V769_D770insGVV (*n* = 1)		1 PR		1 PD
V769_D770insP (*n* = 1)	1 PD			
ORR	6/47 (12.8%)	1/9 (11.1%)	3/18 (16.7%)	2/12 (16.7%)

**Fig. 2:**
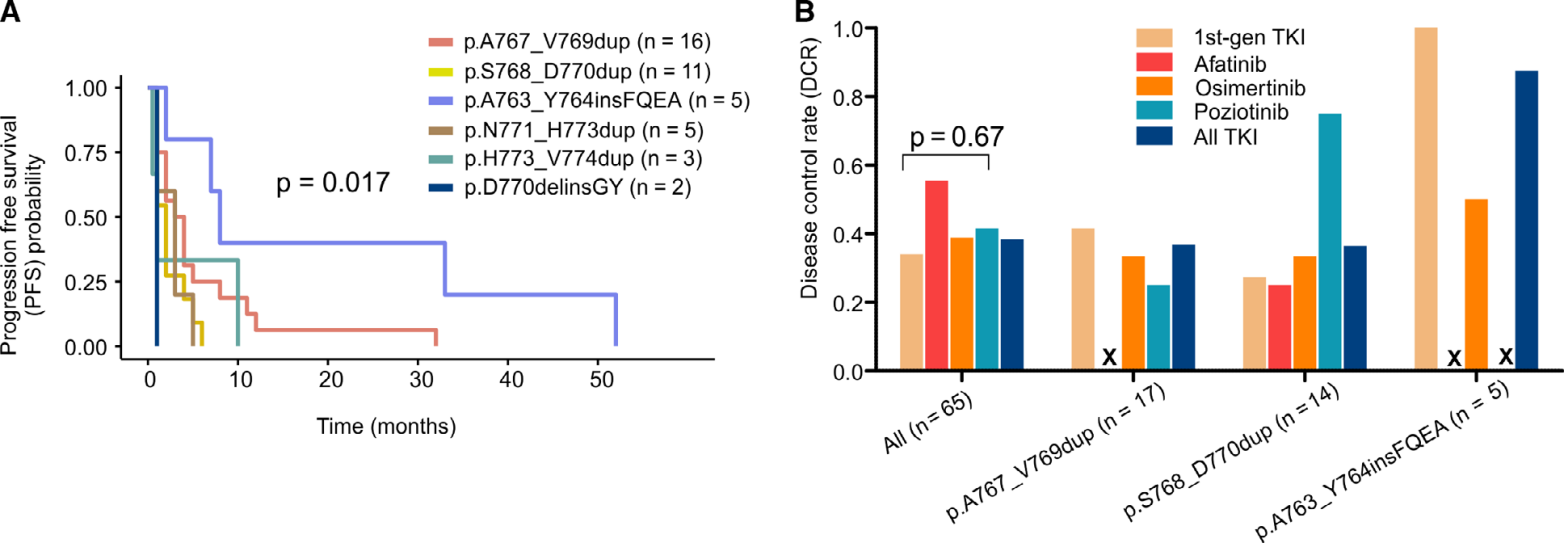
Responses to EGFR inhibitors in different EGFR e20ins. (A) PFS time of different *EGFR* e20ins under TKI treatment. (B) DCR of EGFR inhibitors in different *EGFR* e20ins. ‘x’ indicates zero occurrence.

Progression‐free survival (PFS) analysis of *EGFR* e20ins subtypes revealed that different insertions might react differently to TKI treatment (Fig. 2A, *P* = 0.017). Overall, *EGFR* p.A763_Y764FQEA (*n* = 5) has better PFS than other insertions, and all tumors with this variant were achieved either PR or SD under 1st‐gen TKI treatment (Table 2). One of the two dominant subtypes of e20ins, p.A767_V769dup (*n* = 16), showed considerably varied clinical benefits from TKI among patients. Four patients achieved > 10‐month PFS with the longest to be 32 months, while majority progressed immediately or demonstrated short disease control upon the treatment (Fig. 2A and Table 2). The other major subtype p.S768_D770dup demonstrated limited responses to TKI treatment (Fig. 2A). PFS of these two subtypes is not significantly different from a separate subgroup of patients who received mono or combination cytotoxic chemotherapy as first‐line treatment (*n* = 48, Fig. S3).

**Fig. 3:**
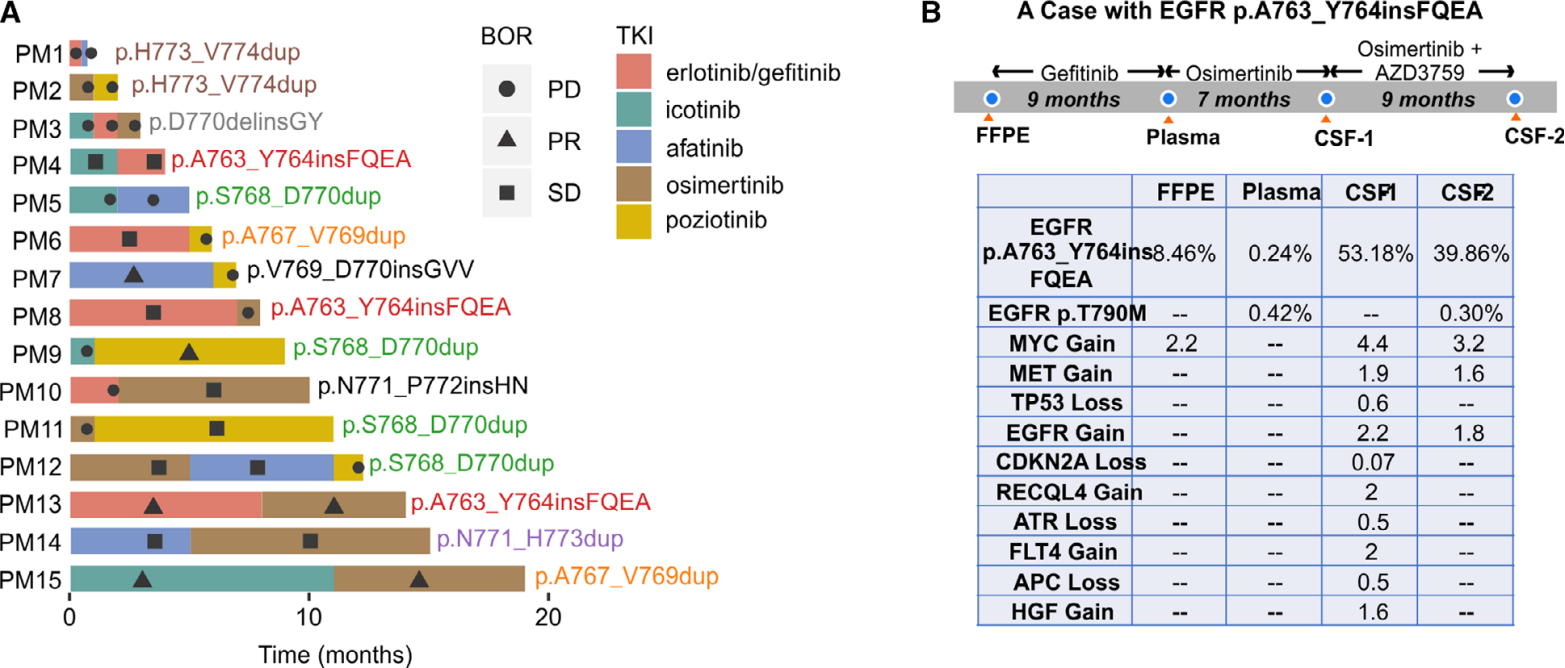
PFS time of different TKIs and the response of patients receiving more than one TKI. (A) The responses and treatment times of patients receiving more than one TKI. Different font colors were used for different insertions. (B) A special case received sequential TKI treatment and the effective control of brain metastasis with combinational use of third‐generation TKI. In the table, MAF and the fold change of CNV were showed. m, month; CSF‐1, the first acquisition of CSF; CSF‐2, the second acquisition of CSF.

Patients with p.D770delinsGY (*n* = 2) developed extremely short PFS compared to others. One of the p.D770delinsGY patients (patient ID: PM4) received gefitinib, icotinib, and osimertinib sequentially, yet none of them has effectively controlled the disease progression, suggesting that this e20ins is insensitive to multiple TKIs (Fig. 3A).

Due to the poor response rate, we also compared DCR of different TKIs. 1st‐gen TKI demonstrated a DCR of 34% (16 out of 47 treatments), while osimertinib (7/18, 38.9%), poziotinib (5/12, 41.7%), and afatinib (5/9, 55.6%) resulted in higher DCR (Figs 2B and S4a,b). For patients with *EGFR* p.S768_D770dup, poziotinib seems to achieve better outcome because three out of four patients have SD or PR at the best response, while only one out of three osimertinib‐treated patients has SD (Fig. 2B, Table 2). It is worth mentioning that as a first‐line therapy, icotinib resulted in long‐term disease control in two patients with the *EGFR* p.D770_N771G mutation (PFS: 39 and 14 months, Fig. S4c).

Fifteen patients received more than one line of TKI, and in most circumstances, reversible and irreversible TKIs were used sequentially (Fig. 3A). As previously reported, erlotinib/gefitinib had a limited effect in treating *EGFR* e20ins patients, showing disease control in only *EGFR* p.A763_Y764insFQEA and p.A767_V769dup patients. Osimertinib showed effective disease control as the second‐line treatment in patients with p.N771_p772insHN, p.A763_Y764insFQEA, p.N771_H773dup, and p.A767_V769dup (patient ID: PM10, PM13, PM14, PM15) (Fig. 3A). Comparatively, when using as the first‐line treatment, its efficacy was limited to p.H773_V774insAH, p.N771_P772insL, and p.S768_D770dup (Figs 3A and S4a). Poziotinib was less effective as a second‐ or third‐line treatment in patients who were previously administered a different first‐ or second‐line TKI (patient ID: PM6, PM7, PM12). We also observed that patients with the same insertions responded differently to sequential treatments. For example, *EGFR* p.A763_Y764insFQEA (*n* = 3), which is the only *EGFR* e20ins reported to be sensitive to different TKIs (Naidoo *et al*., 2015), was found to be responsive to erlotinib/icotinib in two of three patients, while second‐line osimertinib was effective in only one patient, despite both patients acquiring *EGFR* T790M mutations following first‐line treatment. It was also observed that *EGFR* p.H773_774dup (*n* = 2) and p.D770delinsGY (*n* = 1) failed to respond to erlotinib/icotinib or osimertinib/poziotinib. Thus, all such observations suggest varied activities and drug responses for different types of EGFR e20ins, which requires different handling of TKI administration in clinical settings.

### 3.3 A case study

For a patient diagnosed with stage IV NSCLC, he carried *EGFR* p.A763_Y764insFQEA and received sequential TKI treatment (Fig. 3B). Clinical samples including FFPE, plasma, and cerebrospinal fluid (CSF) were obtained to monitor cancer genomics periodically. The first‐line treatment using gefitinib led to 9‐month SD until the presence of resistant mutation *EGFR* p.T790M. Osimertinib as the second‐line treatment resulted in another 7‐month SD until the brain progression. Interestingly, CSF was detected with highly elevated level of *EGFR* p.A763_Y764insFQEA (mutant allele frequency, MAF: 53.18%), and abundant CNV, including copy number gain in oncogenic genes *EGFR*, *MET,* and *MYC*. These unordinary changes might be responsible for the brain metastasis. By adding AZD3759 (Ahn *et al*., 2017), an EGFR inhibitor which is primarily designed to treat brain metastasis of EGFR‐positive NSCLC, the patient’s brain progression was effectively controlled for 9 months. Notably, *EGFR* p.T790M as the acquired resistant mutant to gefitinib was not penetrated to the central nervous system until the progression on osimertinib plus AZD3759 treatment, suggesting it might be not responsible for the brain metastasis.

## 4 Discussion

In this study, by retrospectively analyzing the largest known genetic dataset of lung cancer patients, we uncovered a total of 85 different *EGFR* e20ins and observed disparate responses to EGFR inhibitors in a subset of patients. The low frequency or absence of other accompanying driver mutations in these patients, such as *EGFR* L858R/e19del, *ALK* fusions, *KRAS* mutations, and *MET* amplifications/mutations, among others, is supportive of these insertions being driver mutations, and implicates the potential of targeting such insertions for therapeutic purposes. However, targeting *EGFR* e20ins will be more complicated than targeting the classic *EGFR* mutations, L858R and e19del, as the insertions are too diversified to support a one‐for‐all solution. However, consistent with previous studies (Oxnard *et al*., 2013; Riess *et al*., 2018), this study showed that the two most common *EGFR* e20ins, p.A767_V769dup (25.1%) and p.S768_D770dup (17.6%), comprised ~ 44% of all insertions and can be the main targets in future drug development efforts.

Multiple ongoing clinical trials have begun to test the effectiveness of currently available or novel EGFR TKIs on patients with EGFR e20 insertions, including afatinib and cetuximab (NCT03727724), osimertinib (NCT03414814), poziotinib (Robichaux *et al*., 2018), luminespib (a HSP90 inhibitor) (Piotrowska *et al*., 2018a), and TAS6417 (a novel EGFR TKI) (Hasako *et al*., 2018). However, those studies do not discriminate between the different types of insertions and, thus, provide a blanket treatment for all patients. The preliminary results of a phase II study of poziotinib (Trial No. NCT03066206) in treatment‐naïve patients showed an ORR of 64% in 11 patients (Robichaux *et al*., 2018), but in the most recent update of cohort 1 study in ZENITH20 trial (Trial No. NCT03318939) (BusinessWire, 2019), in which all enrolled patients received systematic treatment before enrollment, ORR of poziotinib was only 14.8%, similar to ORR in our study (16.7%). In our study, poziotinib demonstrated low activity as the second‐/third‐line treatment for patients who had been previously administered a different first‐line TKI, which is most likely due to acquired resistance mutations, which may be insensitive to poziotinib. We also observed a higher DCR of poziotinib to p.S768_D770dup (75%) than to p.A767_V769dup (25%), thus suggesting that different treatment regimens based on the presence of different insertions are necessary for maximizing the efficacy of TKIs. Osimertinib, another promising candidate for treating patients with *EFGR* insertions, has been reported to be effective in suppressing *EGFR* e20ins in sporadic cases (Fang *et al*., 2019a; Piotrowska *et al*., 2018b), specifically for p.S768_D770dup, p.A767_V769dup, p.N771_P772insL, p.D770_N771insG, and p.A763_Y764insFQEA mutations in NSCLC. In our study, more than half of patients used osimertinib after first‐line TKI treatment and demonstrated effective disease control (PR or > 3 months SD) in p.N771_P772insHN, p.S768_D770dup, p.A763_Y764insFQEA, p.N771_H773dup, and p.A767_V769dup patients. However, its first‐line use showed limited effects, especially for two dominant insertions p.S768_D770dup and p.A767_V769dup, implying an unpleasant outcome of its clinical application.

Previous studies have observed the sensitivity of EGFR p.A763_Y764insFQEA to 1st‐gen TKI (Voon *et al*., 2013; Yasuda *et al*., 2013) and 2nd‐gen TKI afatinib (Jorge *et al*., 2018). Homology model simulation suggests that the insertion activates EGFR in a ligand‐independent manner, similar to L858R and exon 19 deletion (Yasuda *et al*., 2013). Here, we confirmed its sensitivity to 1st‐gen TKI and 3rd‐gen TKI osimertinib in a series of patients. One of the patients had controlled disease for more than 2 years by sequential administration of gefitinib, osimertinib, and osimertinib plus AZD3759, suggesting substantial clinical benefits from TKI treatment for this insertion.

Structural analysis of EGFR e20ins offers insight into the mechanism of different TKI responses in EGFR e20ins. The crystal structure of EGFR p.D770_N771insNPG suggests that the insertion leads to constitutive activation of EGFR without increasing the binding affinity to EGFR TKIs (Yasuda *et al*., 2013), which limits its sensitivity to TKI. With the assistant of complex computational structural modeling and molecular dynamics simulation, the investigation of different e20ins revealed diverse binding energy to osimertinib, among which p.A763_Y764insFQEA has the lowest binding energy and therefore the most stable binding of osimertinib (Ikemura *et al*., 2019). Moreover, EGFR p.D770_N771insNPG and p.D770_N771insNPH, despite their similar inserted sequences, showed differential binding energy to osimertinib. These findings, although most are based on *in silico* simulation, unveil the intricate structural and functional changes of different EGFR e20ins and urge the stratified study of each subtype.

Another important finding of this study is that icotinib, a reversible EGFR TKI, achieved a much higher ORR (30.8%) than other TKIs (Fig. S4c). Icotinib demonstrated outstanding clinical efficacy in patients carrying p.A767_V769dup and p.D770_N771insG mutations, but showed no effects in p.S768_D770dup patients. Icotinib has only been approved and marketed in China, and its clinical application resulted in similar treatment outcomes to erlotinib, but was superior to gefitinib (Chen *et al*., 2014; Shi *et al*., 2013). Icotinib has the same anilinoquinazoline scaffold as erlotinib, gefitinib, and lapatinib, but its side chain forms a closed ring structure that increases its hydrophobicity and fat solubility, thus enabling easier transportation across cell membrane and blood–brain barrier (Ni and Zhang, 2016; Yang *et al*., 2017; Zhou *et al*., 2016). Whether icotinib has a higher binding affinity to EGFR e20ins than other TKIs requires the development of a structural compound.

## 5 Conclusions

This study revealed 85 unique *EGFR* e20ins and the most frequent insertions in 547 lung cancer patients, which is helpful for prioritizing drug designing and clinical trials for common insertions. Different insertions demonstrated varied responses to EGFR TKIs, which suggests that the selective use of TKI to treat different *EGFR* e20ins might be useful for improving tumor responses.

## Conflict of interest

XW and XT are the shareholders or employees of Geneseeq Technology Inc., Canada. YS and FW are the shareholders or employees of Nanjing Geneseeq Technology Inc., China.

## Author contributions

YQ, HJ, XW, and YWS contributed to methodology. YQ and HJ investigated the study. YQ, HJ, and XT involved in formal analysis and wrote the original draft. XT and XW curated the data. XT visualized the data. XW and YWS validated the data. FW administrated the project and provided resources. XZ conceptualized; wrote, reviewed, and edited; and supervised the study.

## Supporting information


**Fig. S1.** Frequency and distribution of different EGFR e20ins in the study cohort.
**Fig. S2.** Treatment lines for each TKI in patients who received targeted treatments. 1st‐gen TKI, first‐generation TKI, including gefitinib, erlotinib and icotinib.
**Fig. S3.** Comparing progression free survival (PFS) between TKI treatments and chemotherapy in patients with EGFRp.S768_D770dup (a) and p.S767_D769dup (b).
**Fig. S4.** Progression free survival (PFS) time of different TKIs and best overall response (BOR) of different EGFR e20ins.
**Table S1.** The frequency of accompanying mutations in different EGFR e20ins.
