# Supplementary material for: Variability of EGFR exon 20 insertions in 24 468 Chinese lung cancer patients and their divergent responses to EGFR inhibitors
Source: Mol Oncol. 2020 Jun 15;14(8):1695–704. doi: 10.1002/1878-0261.12710 (PMC7400778; doi:10.1002/1878-0261.12710)
Supplement: Supplementary file 1 — Fig. S1. Frequency and distribution of different EGFR e20ins in the study cohort. Fig. S2. Treatment lines for each TKI in patients who received targeted treatments. 1st‐gen TKI, first‐generation TKI, including gefitinib, erlotinib and icotinib. Fig. S3. Comparing progression free survival (PFS) between TKI treatments and chemotherapy in patients with EGFRp.S768_D770dup (a) and p.S767_D769dup (b). Fig. S4. Progression free survival (PFS) time of different TKIs and best overall response (BOR) of different EGFR e20ins. Table S1. The frequency of accompanying mutations in different EGFR e20ins. [file MOL2-14-1695-s001.docx]

Variability of EGFR exon 20 insertions in 24,468 lung cancer patients and their divergent responses to EGFR inhibitors

Online Supplementary Information


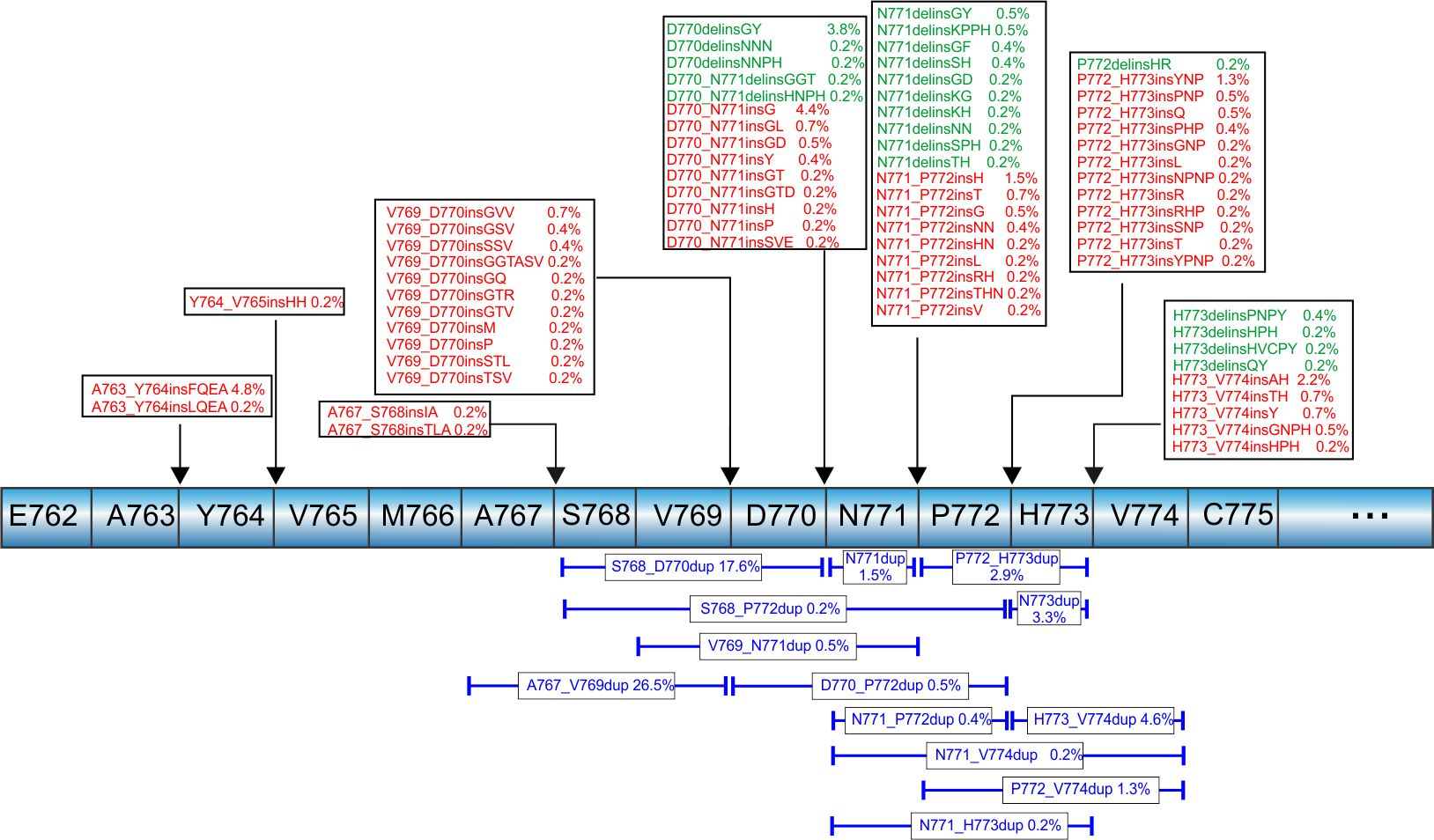
**Fig. S1.** Frequency and distribution of different EGFR e20ins in the study cohort. Red font indicates insertions, green font indicates the coexistence of deletions and insertions, and blue font indicates duplications.


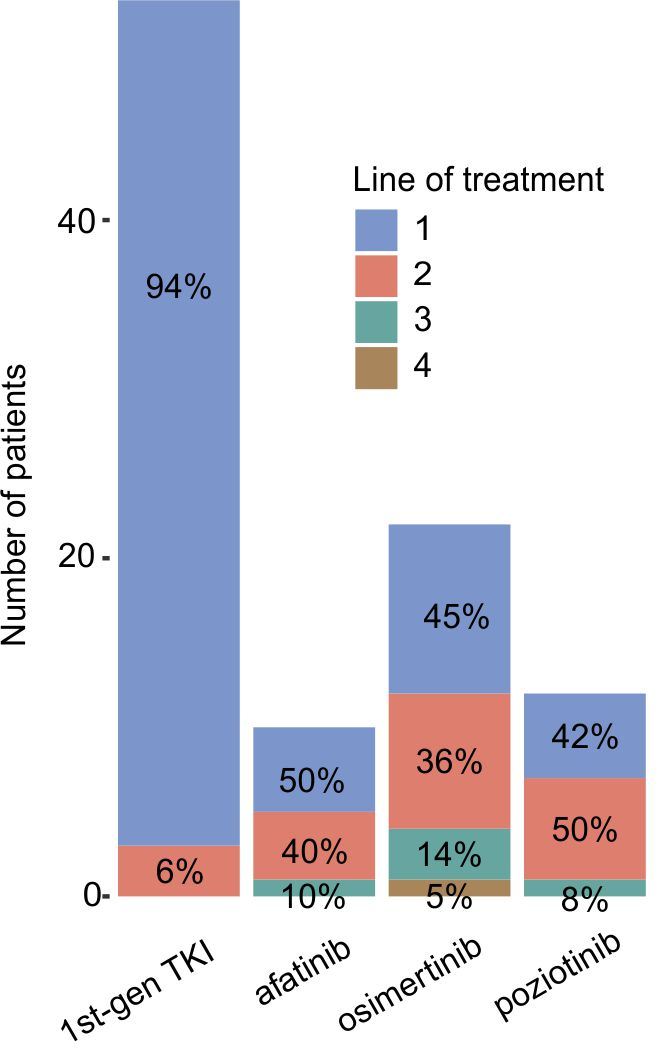


**Fig. S2.** Treatment lines for each TKI in patients who received targeted treatments. 1^st^-gen TKI, first-generation TKI, including gefitinib, erlotinib and icotinib.

**
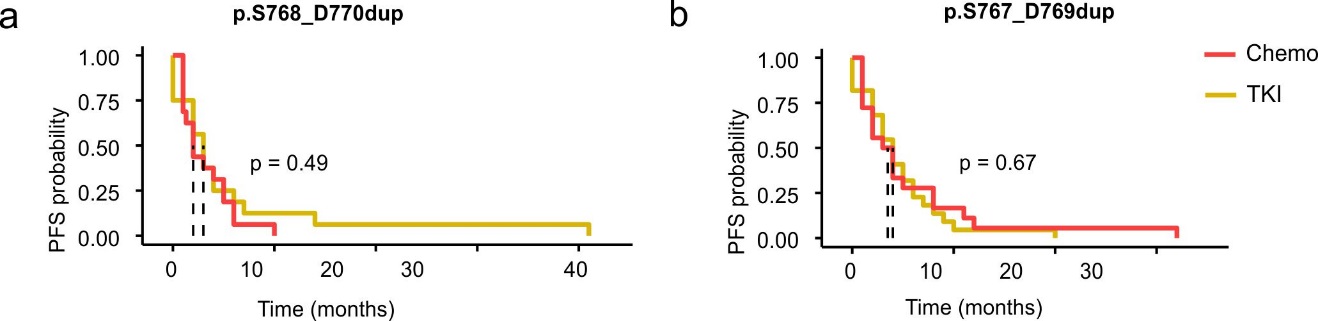
**

**Fig. S3.** Comparing progression free survival (PFS) between TKI treatments and chemotherapy in patients with EGFRp.S768_D770dup (a) and p.S767_D769dup (b).


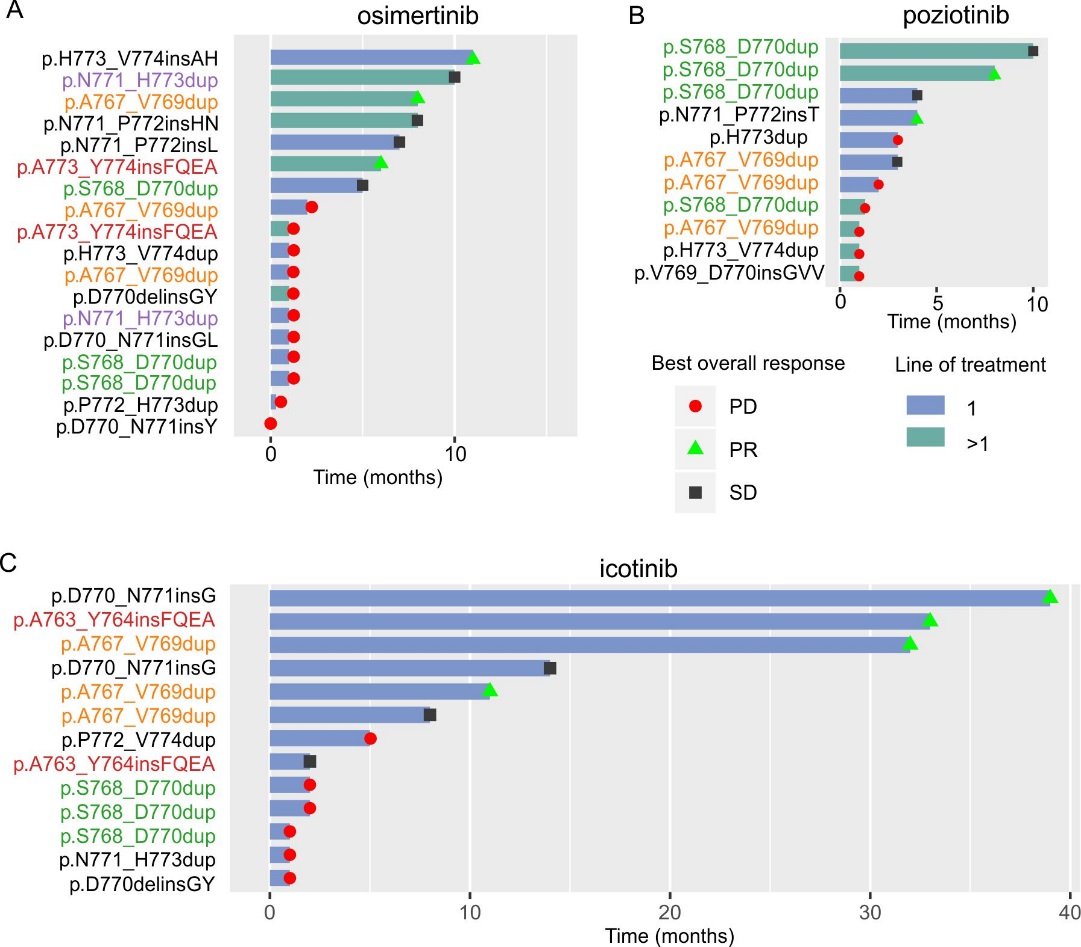


**Fig. S4.** Progression free survival (PFS) time of different TKIs and best overall response (BOR) of different EGFR e20ins.

Supplementary Table 1. The frequency of accompanying mutations in different EGFR e20ins

| **EGFR e20ins** | **No. (%)** | **TP53 (%)** | ***P-value*** | **RB1 (%)** | ***P-value*** | **EGFR amp (%)** | ***P-value*** | **Other primary drivers (%)** | ***P-value*** |
| --- | --- | --- | --- | --- | --- | --- | --- | --- | --- |
| p.A767_V769dup | 145 (26.6%) | 91 (62.8%) | ***0.0325*** | 20 (13.8%) |  | 25 (17.2%) |  | 21 (14.5%) |  |
| p.S768_D770dup | 96 (17.6%) | 45 (46.9%) | ***0.0897*** | 10 (10.4%) |  | 10 (10.4%) |  | 12 (12.5%) |  |
| p.A763_Y764insFQEA | 26 (4.8%) | 16 (61.5%) |  | 1 (3.8%) | ***0.2314*** | 7 (26.9%) | ***0.0738*** | 1 (3.8%) |  |
| p.N771_H773dup | 26 (4.8%) | 16 (61.5%) |  | 5 (19.2%) |  | 4 (15.4%) |  | 3 (11.5%) |  |
| p.H773_V774dup | 25 (4.6%) | 12 (48%) |  | 4 (16%) |  | 4 (16%) |  | 7 (28%) | ***0.0342*** |
| p.D770_N771insG | 24 (4.4%) | 11 (45.8%) |  | 4 (16.7%) |  | 1 (4.2%) |  | 2 (8.3%) |  |
| p.D770delinsGY | 21 (3.8%) | 10 (47.6%) |  | 3 (14.3%) |  | 2 (9.5%) |  | 1 (4.8%) |  |
| p.H773dup | 18 (3.3%) | 14 (77.8%) | ***0.0558*** | 2 (11.1%) |  | 1 (5.6%) |  | 2 (11.1%) |  |
| p.P772_H773dup | 16 (2.9%) | 6 (37.5%) |  | 3 (18.8%) |  | 3 (18.8%) |  | 3 (18.8%) |  |
| p.H773_V774insAH | 12 (2.2%) | 7 (58.3%) |  | 4 (33.3%) | ***0.0551*** | 3 (25%) |  | 4 (33.3%) | ***0.0596*** |
| Others | 137 (25.1%) | 73 (53.3%) |  | 14 (10.2%) |  | 16 (11.7%) |  | 15 (10.9%) |  |
